# Phenolic Compounds from By-Products for Functional Textiles

**DOI:** 10.3390/ma16227248

**Published:** 2023-11-20

**Authors:** Tiago Barros Afonso, Teresa Bonifácio-Lopes, Eduardo Manuel Costa, Manuela Estevez Pintado

**Affiliations:** Laboratório Associado, Escola Superior de Biotecnologia, CBQF—Centro de Biotecnologia e Química Fina, Universidade Católica Portuguesa, Rua Diogo Botelho 1327, 4169-005 Porto, Portugal

**Keywords:** phenolic compounds, by-products, textiles, sustainable dyeing, functional properties, antimicrobial activity, UV protection, antioxidant activity

## Abstract

Textile dyeing is known to have major environmental concerns, especially with the high use of toxic chemicals. The use of alternatives such as natural dyes rich in phenolic compounds has become extremely appealing in order to move towards a more sustainable circular economy. Phenolic dyes have the potential to functionalize textile fabrics with properties such as antimicrobial, antioxidant, and UV protection. Wastes/residues from the agri-food industries stand out as highly attractive sources of these compounds, with several by-products showing promising results in textile dyeing through the implementation of more sustainable and eco-friendly processes. This review presents an up-to-date exploration of the sources of phenolic compounds used in the textile industry over the past two decades, with a primary focus on the functional properties they provide to different fabrics. The research highlights a surge in interest in this theme since 2017, accentuating a noticeable upward trend. Throughout this review, emphasis is given to by-products from the agri-food industry as the sources of these compounds. The reviewed papers lay the foundation for future research, paving the way for exploring the potential of raw materials and by-products in the creation of functional and smart textiles.

## 1. Introduction

The textile industry is known to have major environmental issues of concern regarding the high use of energy, water, and chemicals. The huge amount of chemical usage in its processes makes this industry a major global source of pollution [1]. Synthetic dyes, in particular, pose major challenges as environmental contaminants in textile wastewater due to their non-biodegradable nature, making them difficult to remove from water. Thus, a need has risen to move the textile industry towards a sustainable circular economy [2]. This awareness of eco-safety and increased environmental concern has led to the use of green and sustainable natural dyes as the needed trend in the textile industry [3].

Natural dyes have been used for the coloration of synthetic and natural textile materials since prehistoric times [4]. With the arrival of synthetic dyes in the Industrial Revolution, the use of natural dyes declined to a great extent and practically stopped [4,5]. During the last few decades, researchers’ attention has turned once again towards various aspects of natural dye applications.

Natural dyes are commonly considered eco-friendly as they are obtained from renewable sources and because they are non-toxic, non-carcinogenic, and biodegradable [6]. However, the production and use of natural dyes is not free from environmental concerns as they can be derived from rare, endangered, and threatened (RET) species (e.g., RET plants of Madhya Pradesh, India) or mordant dyes containing heavy metals as part of the dye molecule (e.g., zinc phthalocyanine, acid blue, copper (II) phthalocyanine, lead (II) phthalocyanine, cadmium phthalocyanine) [1,7,8]. According to the latest (4.0) version of the Global Organic Textile Standard (GOTS), dyes obtained from RET species or dyes containing heavy metals are prohibited [9]. Among sources of natural dyes, the use of different raw plant materials and by-products represents an economically and ecologically useful path [10].

Phenolic plant compounds or polyphenols have been gaining interest due to their application as in situ textile dyes as they have colors and have been widely used as natural colorants in the food industry [11]. These compounds are the main plant-derived substances formed by their secondary metabolism. They have always been present throughout human history, long before their formal discovery. Polyphenols have applications in several industries such as the food, pharmaceutical, cosmetic, packaging, and textile industries [12,13,14]. Their use is extremely valuable due to their range of bioactivities, including antimicrobial, antioxidant, anti-proliferative, and anti-inflammatory, among others [11]. They are also responsible for pigmentation and can act as UV protectors, as well as protecting against insects and parasites [15,16]. These compounds can be found in a myriad of diverse matrices including fruits, vegetables, wild plants, etc. An extremely important approach is obtaining phenolic compounds from wastes/by-products of different industries, mainly food processing, but also wood processing and wine-making [17,18,19]. Agri-food bioresidues with no economic value represent a significant percentage of the food processing industries. Consequently, wastes/by-products serve as a natural source of these compounds, being both cheap and abundant while concurrently aiding in the reduction in waste [11].

**Figure 1:**
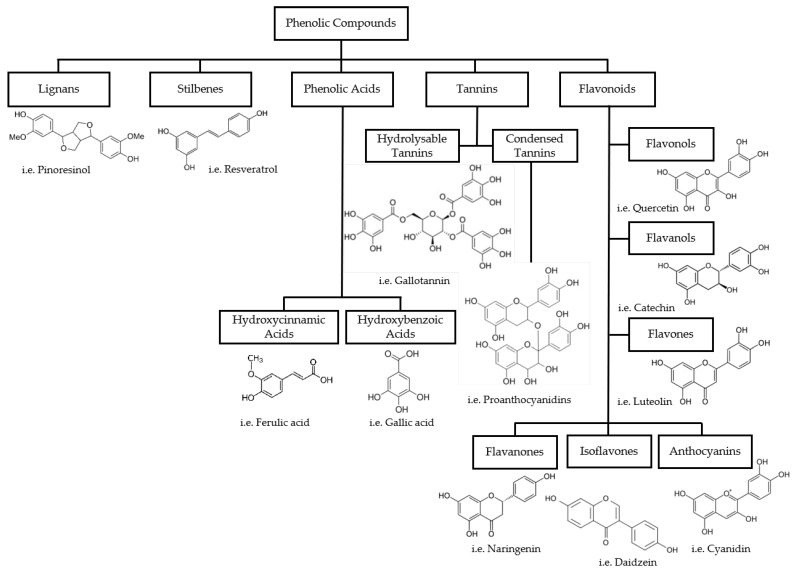
Classification and structural examples of phenolic compounds.

Chemically, phenolic compounds are formed by one or more aromatic rings bonded to one or more hydroxyl (–OH) groups. For this reason, these compounds can be divided into five different groups: phenolic acids, flavonoids, tannins, stilbenes, and lignans [20] (Figure 1).

Phenolic acids are the simplest class of polyphenolic compounds present in foodstuff and their basic structure is characterized by one phenolic ring and a carboxylic acid function. They are derived from two main phenolic compounds, thus being divided into two groups: hydroxybenzoic acids (C6–C1) derived from benzoic acid and hydroxycinnamic acids (C6–C3) derived from cinnamic acid (Figure 1) [20]. The latter is composed of the most common phenolic acids, such as ferulic and caffeic acids, and is responsible for several important bioactivities [20,21].

The most abundant phenolic compounds are flavonoids. They generally comprise a skeleton of carbon atoms (C6–C3–C6) that is built into two benzene rings (A and B), linked by a heterocyclic pyrane ring (C) [11,20]. Depending on the bond between the B and C rings and the substitution patterns of the C ring, they can be divided into six subgroups: flavonols, flavanols, flavones, flavanones, isoflavones, and anthocyanins (Figure 1) [20]. Flavonoids are widespread in several food matrices and are deeply investigated for their range of bioactivities [12].

Tannins are higher-molecular-weight phenolic compounds and are divided into two subgroups: hydrolyzable tannins and condensed tannins, also called proanthocyanidins [20]. They are the most common phenolic compounds found in plant tissues and are responsible for the bitter taste in a variety of fruits [11]. Condensed tannins are oligomers and polymers consisting of two or more monomers of flavan-3-ols units, linked together by bonds between the A rings of the flavanol units and the pyrane rings of other flavanols [20]. Hydrolyzable tannins are mixtures of simple phenols such as ellagic and gallic acids, with a carbohydrate. Gallotannin is an example of a hydrolyzed tannin structure formed from gallic acid (Figure 1) [20]. They are also known to possess a wide range of biological properties [22].

Stilbenes are chemically characterized by two benzene rings linked by a double bond with the structure C6–C2–C6, with the E isomer configuration being the most common [20]. The most known stilbene is resveratrol, with its bioactivities being broadly studied (Figure 1) [20,23].

Lignans are widespread secondary plant metabolites with different chemical structures. However, their common structure is composed of a combination of two phenylpropanoid C6–C3 units, linked by the central carbons of the side chains [20]. One of the main dietary lignans is pinoresinol (Figure 1). Their main properties are related to estrogenic and anti-estrogenic activities [20].

The biological properties of the different types of phenolic compounds make them an extremely interesting group of compounds with potential for use in the textile industry. Beyond their natural coloration ability, these compounds have also been investigated for their potential to impart novel functionalities to textiles, including antimicrobial, antioxidant, ultraviolet (UV) protection, and flame retardance properties, among others [24,25,26,27]. As such, a literature research methodology was completed using the databases Science Direct and Web of Science, and research articles reporting natural dyes containing phenolic compounds applied to textile fabrics were compiled and investigated further. This review dives deeper into the different sources of polyphenols, emphasizing the by-products of the agri-food industry as the main source of these compounds. In addition, the different functional properties provided to textile fabrics are described with ample examples.

## 2. Overview of the Publications

### 2.1. Literature Research Methodology

The research articles about phenolic compounds in the textile industry were searched on the databases Science Direct and Web of Science using the keywords “phenolic compounds” or “polyphenols” and “textiles” from 25 August until 20 September 2023. Therefore, all papers available in these two databases, for the last two decades (2003–2023), which contained the words abovementioned as author-specified keywords in their title or abstract, were considered.

### 2.2. Literature Research Results

**Figure 2:**
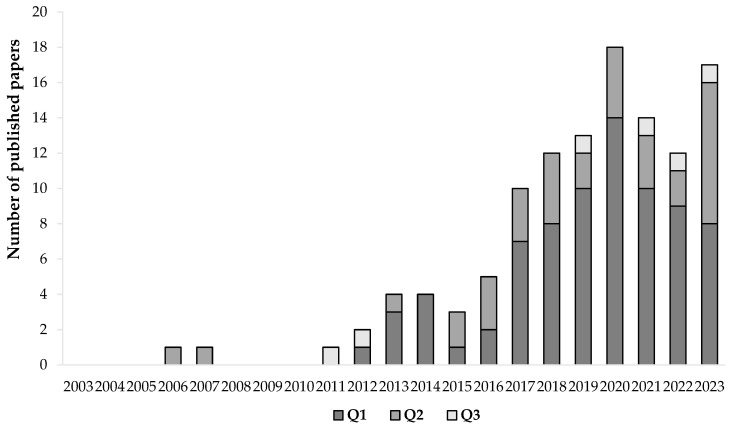
Number of published papers per year since 2003 and respective journal quartiles.

Overall, 12,437 papers were identified and then 8535 papers were excluded for the following reasons: reviews, book chapters, letters, news, patents, meeting papers, reports, papers written in languages other than English, etc. Duplicate papers and records that were not relevant to the topic were excluded after database screening, and 2661 were identified. By screening the titles and abstracts, 2512 papers were removed for not being relevant to the scope of this review. These included papers focusing on dye removal from wastewaters, nanoparticle obtention, enzymatic oligo- and/or polymerization, phenolic compound extraction with no application in textiles, and papers not reporting the presence of phenolic compounds nor reporting dyeing or functional properties provided to textile fabrics, among others. Then, the full texts of 149 papers were reviewed and assessed and as a result, and 117 were included in this review. The number of published papers per year and journal quartile is shown in Figure 2. A full list of the different publishing journals (49) per quartile is shown in Table S1.

The research results revealed only three publications until 2011 while a clear increase in the number of published papers has been observed since 2017, indicating a growing interest in this theme.

Of the selected papers, in addition to dyeing, 68 papers reported antimicrobial/antibacterial properties, 45 reported UV protection, 39 reported antioxidant properties, 4 reported flame retardance properties, and 10 reported other functionalities. Some papers reported more than one activity.

**Figure 3:**
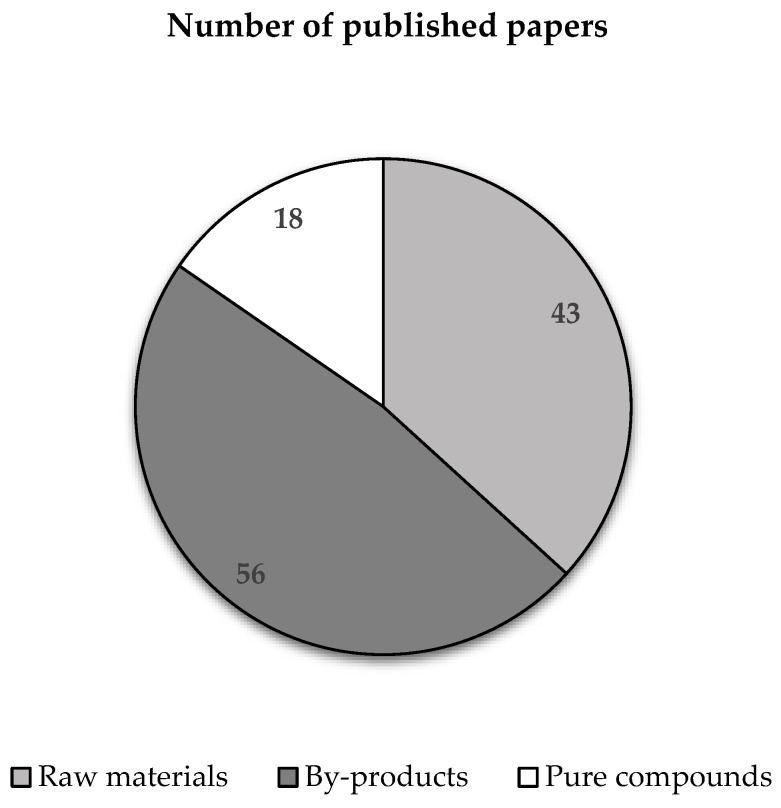
Number of published papers based on the reported sources of phenolic compounds.

The number of papers based on the reported sources of phenolic compounds applied to textile fabrics is shown in Figure 3.

## 3. Sources of Phenolic Compounds for Textile Applications

### 3.1. Pure Phenolic Compounds

Several possible sources of phenolic compounds can be used for textile applications. Pure phenolic compounds that are commercially available have been reported to provide textile fabrics with functional activities (Table 1).

Several of these pure compounds have been directly applied to fabrics [23,28,29,30]. However, in some cases, before being coated onto fabrics, pure compounds suffered some sort of modification, i.e., enzymatic oxidation, crosslinking with other compounds, or they were added to other particles to improve their combined functionality [31,32,33,34,35,36].

There are some potential environmental concerns with the use of phenolic compounds in different industries, especially if their presence is detected in water bodies, as they can be toxic to humans, animals, and microorganisms [37]. However, significant efforts are already being used to remove them from water in case of high levels of contamination. These include conventional methods such as ozonation, activated carbon adsorption, solvent extraction, and more advanced technologies such as the electro-Fenton method, membrane-based separation method, biological methods, photocatalysis, and adsorption and ion exchange [38].

**Table 1:** Commercially available pure phenolic compounds along with their functional properties provided to textile fabrics.

Phenolic Compound	Purpose/Functional Activities	Textile/Fabric	Ref.
Baicalin	Antibacterial, antioxidant	Polyamide	[28,29]
Caffeic acid	Antioxidant, deodorizing, hydrophilicity, UV protection	Nylon, silk, wool	[21]
Catechol	Antimicrobial, antioxidant, UV protection	Cotton, jute, polyethylene terephthalate, wool	[31,32]
Diphenolic acid	Antibacterial, antiviral	Cotton	[39]
*p*-phenylenediamine	Antimicrobial, antioxidant	Cotton, wool, polyethylene terephthalate	[31]
Phloroglucinol	Antibacterial, antioxidant, UV protection	Cotton, jute, wool	[30,32,33]
Pyrogallol	Antibacterial, antioxidant, UV protection	Cotton, jute, wool	[30,32,33]
Pyrocatechol	Antibacterial, antioxidant	Cotton, wool	[30]
Quercetin	Antibacterial, antioxidant	Cotton, polyamide, wool	[29,40]
Resorcinol	Antibacterial, antioxidant, UV protection	Cotton, jute, linen cellulose, wool	[30,32,33,34]
Resveratrol	Antioxidant	Cotton, polyamide	[23]
Rutin	Antibacterial, antioxidant	Cotton, polyamide, wool	[29,40]
Salicylic acid	Antibacterial, UV protection	Linen cellulose	[34]
Tannic acid	Antibacterial, hydrophobicity, UV protection	Cotton, viscose, wool	[35,36,41]

### 3.2. Raw Materials and By-Products

There has been a considerable number of studies reporting a high variability in raw materials or by-products from the agri-food industry as sources of phenolic compounds (Table 2). The common procedure to obtain phenolic compounds from these sources is through the obtention of extracts. In the cases reported here, the extracts obtained were revealed to contain phenolic compounds and had the functionalities of the dyed fabrics attributed to them.

Some of the studies only reported the presence of phenolic compounds through UV–Vis and Fourier-transform infrared spectroscopy (FTIR) analysis [42,43]. Another number of studies reported the quantification of total polyphenols or flavonoids through spectrophotometric methods such as Folin–Ciocalteu, Folin–Denis, or aluminum chloride (AlCl_3_) colorimetric assay [44,45,46]. Finally, beyond total polyphenols or total flavonoids, some studies reported individual phenolic compounds through high-performance liquid chromatography (HPLC) methodologies [47,48,49].

**Table 2:** Raw materials and by-products from the agri-food industry as sources of phenolic compounds and their respective functional activities provided to textile fabrics.

Source	Phenolic Compounds	Purpose/Functional Activities	Textile/Fabric	Ref.
*Acacia auriculiformis* L. bark	Polyphenols, tannins, flavonoids	Dyeing, antibacterial, UV protection	Cotton, silk, wool	[50]
*Acacia nilotica* L. bark	Acacetin, ellagic acid, quercetin	Dyeing, antioxidant	Wool	[51]
*A. nilotica* pods	Polygalloytannin, tannin, quercetin, acacetin, ethyl gallate, digallic acid	Dyeing, antibacterial, antioxidant;	Wool	[52]
*A. nilotica* commercial powder	Quercetin, acacetin	Dyeing, antioxidant, UV protection	Wool	[53]
*Acridocarpus excelsus* bark (by-product)	Polyphenols, flavonoids, condensed tannins, monomeric anthocyanins	Dyeing, antimicrobial, antioxidant	Cotton, silk	[54]
*Alkanna tinctoria* roots	Alkannin, shikonin	Dyeing, antioxidant, UV protection	Wool	[55]
Almond skin (by-product)	Polyphenols	Dyeing	Wool	[17]
*Aloe vera* rinds (by-product)	Polyphenols, flavonoids	Dyeing, UV protection	Silk	[42]
*Amaranthus viridis* plant	Polyphenols, flavonoids	Dyeing, UV protection	Cotton	[56]
Banana floral stem (by-product)	Anthocyanin, anthraquinone, flavonoids, tannin	Dyeing, UV protection	Cotton	[57]
Black tea	Theaflavins, thearubigin	Dyeing	Cotton	[58]
Black tea (Keemun variety) stems and powder waste (by-product)	Theaflavins, theaflavin gallates, catechin	Dyeing, antibacterial, UV protection	Flax	[59]
Buckwheat hull (by-product)	Polyphenols, quercetin, rutin	Dyeing, antibacterial, UV protection	Wool	[60]
*Camellia sinensis* green tea	Catechin, epicatechin, epigallocatechin, epicatechin gallate and epigallocatechin gallate, ferulic acid	Dyeing, antibacterial, antioxidant, UV protection	Cotton, linen, wool	[61,62,63]
Carrot (*Daucus carota* L.) fresh roots (by-product)	Condensed tannins, anthocyanins, hydroxycinnamic acid derivatives	Dyeing, antioxidant	Hemp, wool	[25]
*Cassia alata* flower petals	Polyphenols, flavonoids	Dyeing, antibacterial	Cotton, leather, silk	[64]
Celandine (*Chelidonium majus* L.) fresh leaves and stems	Polyphenols	Dyeing, antimicrobial	Wool	[10]
*Ceriops tagal* bark (by-product)	Polyphenols, flavonoids, condensed tannins, monomeric anthocyanins	Dyeing, antimicrobial, antioxidant	Cotton, silk	[54]
Chebulic myrobalan (*Terminalia chebula*)	Polyphenols, hydrolyzable tannins, chebulagic acid, chebulinic acid, gallic acid, ellagic acid	Dyeing; antibacterial, antioxidant, UV protection	Cotton, wool	[4,55,65]
Chestnut shells (*Castanea crenata*) (by-product)	Polyphenols, ellagic acid, gallic acid, hydrolyzable tannins, ellagitannins, flavonoids	Dyeing, antibacterial, antioxidant, UV protection	Cotton	[66,67]
Chickpea (*Cicer arietinum* L.) husk (by-product)	Polyphenols, tannins, flavonoids	Dyeing, antimicrobial, UV protection	Cotton, silk, wool	[68]
Chinese gallnut (*Galla chinensis*)	Polyphenols, gallotannin, gallic acid, methyl gallate	Dyeing, deodorizing/antibacterial	Cotton, silk, wool	[69,70,71]
Chinese skullcap (*Scutellaria baicalensis*)	Baicalin	Antimicrobial, antioxidant, UV protection	Linen	[28]
Chinese sumac gall (*Rhus chinensis*)-derived gallotannin (commercial)	Gallotannin	Dyeing, antioxidant, antistatic, UV protection	Jute	[72]
Chinese tallow (*Sapium sebiferum* L.) fallen leaves (by-product)	Polyphenols, tannins, flavonoids	Dyeing, antibacterial, antioxidant, UV protection	Wool	[73]
*Cinnamomum camphora* tree fallen leaves (by-product)	Polyphenolics, flavonoids, tannins, anthocyanins, quercetin, kaempferol, rutin	Dyeing, antibacterial, UV protection	Wool, silk	[74,75]
Cooper plant (*Acalypha wilkesiana*) leaves	Polyphenols, gallic acid, quercetin, tannins, corilagin, geranin	Dyeing	Cotton	[76]
Cork industry by-products: cork-cooking wastewater (CCW), expanded black cork condensate (EBCC)	CCW—polyphenols, tannins, flavonoids, anthraquinones; EBCC—polyphenols	Dyeing, antibacterial	Cotton, wool	[77]
*Croton urucurana* Baill. bark	Tannins	Dyeing, UV protection	Wool	[78]
Curry plant (*Helichrysum italicum* Roth) flowers	Pyrogallol, chlorogenic acid, gallic acid, cynarin, naringenin, pinocembrin, chrysin, coumarin	Dyeing, UV protection	Cotton, flax, polypropylene	[15,79]
Date palm pits *(Phoenix* *dactylifera)*	Polyphenols, gallic acid, protocatechuic acid, *p*-hydroxybenzoic acid, vanillic acid, caffeic acid, *p*-coumaric acid, ferulic acid	Dyeing	Cotton	[80,81]
*Delonix regia* flowers	Quercetin, gallic acid	Dyeing, antioxidant, UV protection	Wool	[55]
*Dioscorea cirrhosa* tuber (by-product)	Condensed tannins	Dyeing, antibacterial, antioxidant, flame retardance	Silk	[82]
Eucalyptus (*Eucalyptus camaldulensis*) leaves	Quercetin, rutin, ellagic acid	Dyeing	Cotton, wool	[40]
Eucalyptus (*E. grandis*) liquid residue from lumber steaming	Condensed tannins, quercetin, ellagic acid, rutin	Dyeing	Cotton, nylon, wool	[19]
Feijoa peel (by-product)	Procyanidin B1, epicatechin, quercetin-3-galactoside, gallic acid, quercetin	Dyeing, antibacterial, antistatic, antioxidant, hydrophilicity, insect resistance, UV protection	Wool	[16]
Fennel (*Foeniculum vulgare*) leaves (by-product)	Polyphenols, flavonoids (kaempferol, quercetin)	Dyeing	Cotton	[83]
Ginkgo (*Ginkgo biloba* L.) tree leaves (by-product)	Polyphenols, flavonoids (quercetin, quercitrin, rutin)	Dyeing, antibacterial	Wool	[24]
*Glochidion eriocarpum* Champ leaves	Ellagic acid, gallic acid, quercetin	Dyeing, antibacterial, UV protection	Cotton	[84]
Grape seed proanthocyanidins (commercial)	Proanthocyanidins	Dyeing, antibacterial, antioxidant, anti-pilling, antistatic, flame retardance, UV protection	Silk, cashmere, cotton	[18,27,85]
Groundnut (*Arachis hypogaea*) testa (by-product)	Polyphenols, tannins, flavonoids	Dyeing, antibacterial, UV protection	Cotton	[86]
Henna (*Lawsonia inermis*)	Polyphenols, tannin, gallic acid	Dyeing, antibacterial, antioxidant, UV protection	Linen, wool	[26,52]
*Hibiscus* flowers (*Hibiscus sabdariffa*)	Hydroxy citric acid, hibiscus acid, chlorogenic acid, hydroxy coumarin, N-feruloyl tyramine, rutin, apigenin, myricetin quercetin, anthocyanins	Dyeing, antimicrobial, antioxidant, UV protection	Cotton, wool	[87]
*Houttuynia cordata* perennial herb	Polyphenols, flavonoids (quercitrin)	Dyeing, antioxidant	Polyurethane nanofibers	[46]
*Hypercium scabrum* L. plant	Polyphenols, tannins, flavonoids	Dyeing	Wool	[88]
*Kalanchoe pinnata* leaves	Polyphenols, tannins, flavonoids	Dyeing, antibacterial, antioxidant	Milk, silk, soya, wool	[89]
Immature pine cone	Tannins	Dyeing, deodorizing/antibacterial	Cotton, silk, wool	[90]
Lotus leaf	Polyphenols, flavonoids	Dyeing, hydrophobicity	Polyester	[91]
*Lycium ruthenicum* Murray dried fruits	Anthocyanins	Dyeing, antibacterial, antioxidant	Wool	[92]
Madder (*Rubia tinctorum* L.) roots	Anthraquinones (purpurin, xantho-purpurin, rubiadin, pseudopurpurin, munjistin, lucidin)	Dyeing	Polyester	[1]
Madder powder	Alizarin, ruberythric acid, rubiadin, purpurin	Dyeing, antibacterial	Wool	[93]
Mango (*Manifera indica* L.) seed kernel (by-product)	Ferulic acid, gallic acid, cinnamic acid, vanillin, tannin, mangiferin	Dyeing, antibacterial, antistatic, antioxidant, hydrophilicity, insect resistance, UV protection	Cotton, wool	[16,49]
Mango leaves (by-product);	Gallic acid, mangiferin, iriflophenone	Dyeing	Cotton	[94]
Mango leaves cv. Kent (by-product)	Gallic acid, mangiferin, iriflophenones, quercetin	Dyeing, antibacterial, antioxidant	Polyester	[95]
Mangrove bark (by-product)	Phenolics, catechin, epicatechin, epigallocatechin, epigallocatechin gallate	Dyeing	Cotton	[96]
*Melia azedarach* bark (by-product)	Polyphenolics, flavonoids	Dyeing, anti-moth, fluorescence, UV protection	Wool	[97]
Mushroom *(Cortinarius semisanguineus)*	Anthraquinones	Dyeing	Cellulose fabrics	[98]
Naturally colored cottons (brown)	Condensed tannins	Antibacterial	Cotton	[99]
Oak bark (by-product)	Gallotannin, ellagitannin, quercetin, quercetin-3-oglucoside	Dyeing, antimicrobial, UV protection	Silk	[6]
Olive mill wastewater	Luteolin, quercetin, apigenin	Dyeing	Wool	[100]
Onion (*Allium cepa* L.) skin (by-product)	Condensed tannins, anthocyanins, quercetin, quercetin derivatives, protocatechuic acid	Dyeing, antioxidant	Hemp, wool	[25,101]
Onion (*A. cepa* cv. Settonia) skin (by-product)	Quercetin aglycone, quercetin glycosides	Dyeing	Cellulose fabrics	[98]
Onion (*A. cepa* cv. Red Baron) skin (by-product)	Quercetin, taxifolin, cyanidin, delphinidin, peonidin	Dyeing, UV protection	Cotton	[102]
Onion (*A. cepa* cv. Dorata di Parma) skin (by-product)	Protocatechuic acid, vanillic acid quercetin, ellagic acid, isorhamnetin	Dyeing, antibacterial, antioxidant, UV protection	Wool	[103]
Orange peel (by-product)	Phenolic colorants; *p*-coumaric acid, vanillic acid, gallic acid, caffeic acid, ferulic acid, catechin, sinensetin, nobiletin	Dyeing, antimicrobial, antioxidant, insect resistance, UV protection	Viscose, wool	[104,105]
*Papaver rhoeas* flower	Polyphenols, flavonoids	Dyeing	Cotton, wool, viscose	[106]
Peanut (*Arachis hypogaea* L.) red skins (by-product)	Homovanillic acid, protocatechuic acid, gallic acid, procyanidin B4, catechin, kaempferol	Dyeing, antibacterial, antioxidant, UV protection	Viscose	[107]
Peanut roasted red skins (by-product)	Polyphenols, tannins, flavonoids	Dyeing, UV protection	Cotton, silk, wool	[108]
*Pelargonium graveolens* stems and leaves (by-product)	Polyphenols, flavonoids, condensed tannins	Dyeing	Wool	[109]
Pineapple (*Ananas cosmosus*) peel (by-product)	Polyphenols, flavonoids	Dyeing, antibacterial, antioxidant, UV protection	Wool	[110]
Pomegranate peel (*Punica granatum* L.) (by-product)	Polyphenols, punicalagin, ellagic acid, gallic acid, tannins, flavonoids, quercetin, N-methyl granatonine	Dyeing, antimicrobial, UV protection	Cotton, hemp, polyamide, wool	[43,44,111,112,113]
*Portulaca oleracea* L. plant	α-Linolenic acid, catechin, kaempferol, *p*-coumaric acid, quercetin, tannic acid	Dyeing, antibacterial, UV protection	Cotton	[114]
Purple-fleshed sweet potato	Anthocyanins (cyanidin, peonidin), phenolic acids	Dyeing, antibacterial, antioxidant	Cotton, silk, wool	[115,116]
*Pterocarpus santalinus* tree waste (by-product)	Flavonoids (isoflavones, pterocarpans, santalins)	Dyeing, antibacterial, antioxidant	Wool	[117]
Quince (*Cydonia oblonga*) leaves (by-product)	Flavonoids, condensed tannins	Dyeing, antimicrobial	Wool	[118]
Red pepper (*Capsicum annum* L.) seeds, skin leftovers, and stems (by-products)	Polyphenols, flavonoids	Dyeing, antibacterial	Wool	[119]
*Reseda luteola* L. plant	Polyphenols, flavonoids (7-O-glucoside luteolin)	Dyeing, antibacterial	Wool	[47]
*Rhizophora mucronata* bark (by-product)	Polyphenols, flavonoids, condensed tannins, monomeric anthocyanins	Dyeing, antimicrobial, antioxidant	Cotton, silk	[54]
Rice straw (by-product)	Polyphenols, flavonoids	Dyeing, antibacterial, flame retardance, UV protection	Wool	[120]
Saffron (*Crocus sativus* L.) flower waste (by-product)	Polyphenols, flavonoids	Dyeing, antioxidant	Cotton	[121]
Saffron petals	Miricetin, quercetin, delphinidin, petunidin, kampferol	Dyeing, antibacterial	Wool	[93]
Sage (*Salvia officinalis* L.) dried leaves and stems (by-product)	Hydroxycinnamic acid derivatives, luteolin	Dyeing, antioxidant	Hemp, wool	[25]
*Scrophularia striata* aerial parts (by-product)	Cinnamic acid, caffeic acid, vanillin, trans-ferulic acid, hesperidin, rosmarinic acid; quercetin, nepitrine, isorhamnetin	Dyeing, antibacterial	Wool	[122]
*Solanum nigrum* plant	Polyphenols, flavonoids	Dyeing, UV protection	Cotton	[56]
Sorghum husk (by-product)	Polyphenols, flavonoids (apigeninidin, luteolinidin)	Dyeing, UV protection	Cotton, wool	[123,124]
Spent coffee grounds (by-product)	Polyphenols, tannins, catechins, flavanols, chlorogenic acid, caffeoylquinic acid	Dyeing, antibacterial, antioxidant, UV protection	Silk, wool	[125]
Sweet potato (*Ipomoea batatas*) leaves (by-product)	Polyphenols, tannins, flavonoids	Dyeing, antibacterial, UV protection	Cotton, nylon, polyester, silk, wool	[126]
*Tamarix aphylla* (L.) Karst. leaves	Apigenin, caffeic acid, ellagic acid, isorhamnetin, luteolin, *p*-coumaric acid, syringic acid; quercetin, tamarixetin	Dyeing	Cotton	[48]
Tea polyphenols (commercial)	Catechin, gallocatechin, catechin gallate, gallocatechin gallate	Dyeing, hydrophobicity, UV protection	Cotton, silk, wool	[3,41]
Tea stem waste (by-product)	Polyphenols	Dyeing, antibacterial, antioxidant, flame retardance	Silk	[127]
*Terminalia arjuna* fruits	Polyphenols, tannins, flavonoids (lucenin, quercetin)	Dyeing	Cotton, nylon, silk	[128]
*T. arjuna* powder (commercial)	Ellagic acid, baicalein	Dyeing, antioxidant, UV protection	Wool	[53]
*Thespesia populnea* fruits	Polyphenols, tannins, flavonoids (lucenin, quercetin)	Dyeing	Cotton, nylon, silk	[128]
Thyme (*Thymus vulgaris* L.) dried leaves and stems (by-product)	Hydroxycinnamic acid derivatives, luteolin	Dyeing, antioxidant	Hemp, wool	[25]
Thyme essential oil/beeswax matrix emulsion	Polyphenols, flavonoids	Dyeing, antibacterial	Cotton	[129]
Vine leaves (by-product)	Polyphenols, flavonoids	Dyeing, antibacterial	Viscose	[45]
Walnut (*Juglans regia* L.) green husks (by-product)	Polyphenolics, tannins, punicalagin	Dyeing, antimicrobial	Wool	[112]
Walnut shells	Tannic acid, juglone, gallic acid	Dyeing, antimicrobial	Wool	[130]
Watermelon rind (by-product)	Anthocyanin, anthraquinone, hydrolyzable, condensed tannins, flavonoids, quercetin	Dyeing, UV protection	Cotton	[57,94]
Wild lavender (*Lavandula stoechas* L.)	Hydroxycinnamic acid, flavonoids, coumarins, anthraquinones	Dyeing, UV protection	Cotton, flax	[15]
Wild madder (*Rubia peregrina* L.)	Hydroxycinnamic acid, flavonoids, coumarins, anthraquinones	Dyeing, UV protection	Cotton, flax	[15]
*Woodfordia fruticosa* adventitious roots (by-product)	Polyphenols, flavonoids, condensed tannins, monomeric anthocyanins	Dyeing, antimicrobial, antioxidant	Cotton, silk	[54]
*Xylocarpus granatum* bark (by-product)	Polyphenols, flavonoids, condensed tannins, monomeric anthocyanins	Dyeing, antimicrobial, antioxidant	Cotton, silk	[54]

The high quantity of by-products and wastes generated by the agri-food industries (frequently reaching up to 50%) create safe disposal issues and contribute to negative environmental impacts [131,132]. According to the Food and Agriculture Organization of the United Nations (FAO) report, the carbon footprint of vegetables has significantly increased mainly due to large volumes of waste, while fruit wastage has emerged as the major blue water hotspot, especially in industrialized countries in Asia and Europe [133]. For example, the processing waste generated by citrus fruit production is traditionally used as animal feed or directly discarded as waste without any treatment, resulting in serious environmental problems. Given the strong antimicrobial activity of citrus essential oils, concerns emerge regarding the inhibition of natural soil microflora [134]. For these reasons, the valorization of these by-products has become an utmost necessity. Although some of the generated by-products can be considered unavoidable, others can be utilized in different areas, including the textile industry. The valorization of these by-products is a serious alternative to establishing sustainable developments and to reducing environmental problems related to the textile industry [44]. Thus, of the identified sources of phenolic compounds, by-products are one of the most appealing.

Several by-products were identified in this literature review, including skins/peels, barks, seeds, leaves, stems, roots of different plants and fruits, and even wastewaters resulting from their processing. These by-products can represent a large percentage of the agri-food processing industries resulting in millions of tons of waste. For example, orange or pomegranate peels can represent roughly 20–30% or up to 40% of a whole fruit, respectively [104,135]. Skins from almonds can represent up to 8% of a total shelled almond weight [17]. The production of onion, the second most abundant horticultural crop in the world, generates half a million tons of biowaste (skins) in Europe alone [102]. In some extreme cases, such as in the *R. luteola* plant, about 300 g of by-products (stems, roots, and grains) are discarded in order to harvest just 1 g of leaves [47].

In the few instances where by-products were liquid in nature, direct dyeing with the by-product could be achieved [77,100]. However, in most cases, phenolic compounds from solid by-products needed to be extracted to be applied to textile fabrics. Different extraction conditions were used, but almost all studies utilized green, sustainable, and eco-friendly procedures to achieve the whole of the dyeing process. These sustainable approaches included some of the following practices: (i) reducing the use of organic solvents while using water as the main extracting solvent or other green extraction procedures; (ii) minimizing or eliminating the use of toxic metal salt mordants; (iii) using bio-sourced mordants; (iv) directly applying extracts without any type of mordants; (v) applying extracted dyes to fabrics by eco-friendly techniques; (vi) monitoring the biodegradability of the generated wastewaters; and (vii) using life cycle assessments to determine the environmental impacts associated with the dyeing processes [1,4,48,64,72,86,96].

All of these practices used either separately or as a combination of each other allow for the sustainable and eco-friendly dyeing of textile fabrics with polyphenolic dyes.

## 4. Functional Properties of Phenolic Dyed Textile Fabrics

Functional finishing is always an exciting treatment in which fabrics can be given interesting performance/functional properties during textile processing. Natural dyes rich in polyphenols can directly provide desirable finishing properties during the dyeing process without the need for a separate finishing. Due to different functional groups, these dyes can form different interactions between the dye and the fabric, allowing for different functional properties to be achieved [20]. While some dyes only report one function, most of them can be responsible for different functionalities at the same time. The two main reported functionalities provided to textile fabrics are antimicrobial/antibacterial and UV protection, followed by antioxidant performance. To a lesser extent, other functionalities have also been reported, i.e., flame retardance, hydrophobicity, insect resistance, and moth proofing.

### 4.1. Antimicrobial/Antibacterial

The human body is constantly being exposed to a variety of microorganisms such as bacteria and other microbes. While clothing fabrics usually cover a significant part of the human skin, they inherently lack effective antimicrobial resistance. On the contrary, they have been recognized as a medium for supporting bacterial growth and proliferation [70]. For instance, some fabrics such as wool, due to its proteinaceous nature, under ambient conditions of moisture and temperature, can serve as a growth promoter for a large number of bacterial strains [24]. Subsequently, this bacterial growth can lead to the discoloration and degradation of textile fabrics or more importantly, to an increased risk of dermal infection and allergic responses [24]. For these reasons, providing antimicrobial/antibacterial properties to textile fabrics becomes crucial.

Different methodologies have been used to test the antimicrobial/antibacterial properties of textile fabrics dyed with natural dyes rich in polyphenols. These include methodologies such as zone of inhibition and agar diffusion tests, percentage reduction assays, spectrophotometric assays, and minimum inhibitory concentration determinations, among others [24,47,73,111]. These have usually been performed in accordance with standardized methodologies, i.e., AATCC TM 100 [136], AATCC TM 90:2016 [137], ASTM E2149 [138], ISO/DIS 20743 [139], and GB/T 20944.3-2008 [44,47,70,82,111,140].

The two most tested microorganisms for evaluating antimicrobial/antibacterial properties of dyed fabrics are bacterial strains such as *Escherichia coli* (Gram-negative) and *Staphylococcus aureus* (Gram-positive) [6,28,29,68]. Other commonly used bacteria are *Klebsiella pneumoniae*, *Pseudomonas aeruginosa*, *Bacillus subtilis*, and *B. cereus*, while *Candida albicans* is the most commonly selected fungus [54,71,73,125,130].

The natural dyes reported here (Table 2) possess polyphenols in their constitution and the antimicrobial functionality of the dyed textiles is usually attributed to these compounds. Phenolic compounds attach onto fabrics by forming a complex, and when microorganisms come into contact with the fabrics, these compounds can disrupt their enzyme production which eventually results in the death of the cell. For instance, several dyed fabrics were able to achieve a great percentage of inhibition against bacteria. These include dyed fabrics with pure phenolic compounds such as pyrogallol, phloroglucinol, pyrocatechol, and resorcinol, which were able to inhibit >99.9% of *S. aureus* and >99.6% of *E. coli* in cotton and wool fabrics [30]. A dye obtained from Keemun black tea, with theaflavins as the major polyphenol in its composition, was able to inhibit >99.9% of *S. aureus* in flax fabric [59]. Aqueous chestnut shell extracts, having condensed tannins and gallic and ellagic acids, were able to achieve percentage inhibitions of >99.9% against *S. aureus* and *K. pneumoniae* in cotton fabric [66,67]. Several other dyes were also able to achieve over 90% inhibition against microbes. Fang et al. [126] reported percentage inhibitions of >98% against *S. aureus* and *E. coli* with an extract containing tannins and flavonoids obtained from *I. batatas* leaves, in wool, cotton, nylon, polyester, and silk fabrics. Interestingly, these fabrics maintained very good inhibition percentages (>84%) even after 30 wash cycles. Other natural dyes were able to maintain some of their antimicrobial activity after washing. Wool fabric dyed with an extract obtained from rice straw showed >98% inhibition against *S. aureus* and >80% was maintained after 20 washing cycles [120]. In addition, cotton fabric dyed with *G. ericarpum* leaf extracts showed >80% inhibition against *E. coli* after five washing cycles [84].

In some cases where the extract itself did not display strong antimicrobial activity, a combination with different mordants was able to enhance this functionality. For example, Sadeghi-Kiakhani et al. [112] showed that wool fabrics dyed with extracts from pomegranate peels and walnut green husks displayed around 65% inhibition against *E. coli* and *S. aureus*. In the same study, when the wool fabric was pretreated with Ag or Cu before being dyed with the extract, it was able to achieve >99.9% inhibition. In addition, after 10 washing cycles, the fabric maintained >91% inhibition against both bacteria. Thus, the application of mordants (i.e., aluminum, tannic acid, chitosan), crosslinking, or other surface modifications with cationization or by applying a biopolymer are responsible for increasing functionality and providing washing stability [33,61,62,93,116].

### 4.2. UV Protection

Several diseases are directly linked to the exposure of skin to solar UV radiation, such as freckles, sunburns, and in extreme cases, skin cancer [15]. With the harmful changes occurring in our climate, these problems are becoming more common and exacerbated, resulting in the need for protection against UV radiation. Solar UV light radiation contains three parts: UV-A (400 − 315 nm), UV-B (315 − 290 nm), and UV-C (290 − 200 nm). The main concern regarding damage to human skin is UV-A as most of the UV-B and UV-C are filtered by the ozone layer [105]. 

Different synthetic UV absorbers are currently available for the textile industry, but there is an obvious need to search for more sustainable alternatives. The UV protective property of dyed fabrics is typically analyzed using their ultraviolet protection factor (UPF) as an indicator. This UV protective analysis is usually completed in accordance with standardized methods, i.e., AATCC 183 [141], GB/T 18830-2009 [142], AS/NZS 4399:1996 [143], and EU standard 13758-2001 [28,75,78,120,144]. The UPF scale is the following: 15–24, good; 25–39, very good; and 40–50+, excellent.

Textile fabrics by themselves have poor UPF values (<15) and thus, cannot offer sufficient UV protection [68]. The application of natural dyes rich in polyphenols onto textile fabrics can significantly increase their UPF. Several of the polyphenolic extracts mentioned in Table 2 were able to confer textile fabrics with excellent (50+) UPF capability. For instance, cotton fabric dyed with *S. nigrum* or *A. viridis* displayed UPF values of 60+ and 100+, respectively. This excellent UPF was attributed to the presence of polyphenols and flavonoids as *A. viridis* extract had a higher content of these compounds [56]. Flavonoids are known for their capacity as UV absorbers, and present wavelength selectivity for UV-B which may prevent the accumulation of UV-B-induced damage [56]. The excellent UV protective properties (100+ UPF) of wool fabrics dyed with orange peel extracts were reported to not only be due to the absorbability of UV rays by colored phenolic components, but also by the presence of other colorless phenolic compounds [104]. Cotton fabric, dyed with a natural dye obtained from groundnut testa, revealed an excellent UPF of 50+. This UV protective functionality was attributed to the presence of tannins, phenols, and flavonoids as these compounds exhibit free radical scavenging capability [86]. Other fabrics dyed with extracts rich in flavonoids and tannins have also reported a 50+ UPF, such as wool fabric dyed with rice straw and *A. auriculiformis* extracts [50,120]. Guo et al. [18], reported that the excellent UV protection of cotton fabric dyed with grape seed extract was due to the high number of aromatic rings present in proanthocyanins.

The same dye can provide different UPF values on different fabrics. For instance, cotton dyed with an extract obtained from roasted peanut skin showed <15 UPF, while silk and wool fabrics dyed with the same extract showed 50+ UPF [108].

Although natural dyes improve the UV protective properties of fabrics, there are some limitations associated with them. After long exposure to UV rays as well as several washing steps, this protection might be reduced or even lost. Otaviano et al. [43] reported a good UPF (25) for cotton dyed with pomegranate peel extract, but after 10 washing cycles, no UV protection was detected. To address this issue, mordants can be used. In this same study, with the combination of natural dye with Fe (II), the fabric was able to maintain a good UPF throughout the washing cycles [43]. In addition, the use of chitosan as a mordant allowed cotton fabric dyed with *G. ericarpum* to maintain a UPF of 30 after five repeated washing cycles [84]. Nevertheless, other natural dyes were able to provide their UV protective function to textiles after laundry cycles without any mordant. For example, cashmere dyed with grape seed proanthocyanins, wool fabric dyed with Sorghum husk extract, and silk fabric dyed with *A. vera* rind extract all maintained an excellent (50+) UPF even after 20, 30, and 25 washing cycles, respectively [42,85,124].

### 4.3. Antioxidant

Free radicals present in the atmosphere are considered a major cause of several specific human diseases, making antioxidant activity a subject of intense interest. However, the antioxidant activity of textiles has not attracted proper attention in the past, while in reality, clothes with an antioxidant function can provide the skin with protection against free radicals that are responsible for skin aging [18]. Phenolic compounds present in natural dyes are well known for their antioxidant properties, being considered their most effective feature. The antioxidant activity of these compounds is mainly owed to their redox properties, which help to captivate and neutralize free radicals [105]. For instance, phenolic acids usually display antioxidant activity by trapping free radicals, while flavonoids can scavenge them [29]. Thus, when applied to clothing materials, phenolic compounds will help protect the skin from various types of damage by slowing the effects of free radicals [33].

The two main reported methodologies for evaluating the antioxidant activity of dyed fabrics with natural dyes are 1,1-diphenyl l-2-picrylhydrazyl free radical (DPPH^•^) and ABTS radical cation (ABTS^•+^) scavenging activities [29,46,95]. Results are typically shown in terms of percentages of inhibition.

Undyed textile fabrics have a poor ability to catch free radicals and poor antioxidant function [87,127]. Several natural dyes reported in Table 2 were able to provide antioxidant properties to different fabrics. Linen fabric dyed with *S. baicalensis* showed 84% free radical scavenging activity. This was due to the presence of the phenolic compound baicalin [28]. Polyamide fabric dyed with quercetin showed above 90% free radical scavenging activity [29]. Guinot et al. [25] reported excellent antioxidant activity in hemp and wool fabrics dyed with *S. officinalis*, *T. vulgaris*, and *A. cepa* extracts. This was attributed to the high content of flavonoids and hydroxycinnamic acid derivatives in their composition. Different natural dyes obtained from by-products were also able to functionalize textile fabrics with high antioxidant activity. Among others, wool fabric dyed with *S. sebiferum* fallen leaf extract, silk fabric dyed with tea stem waste extract, and viscose fabric dyed with orange peel extract showed antioxidant activity above 90% [67,73,85,105,121,127].

Several dyed fabrics were also able to maintain some of their antioxidant activity after washing cycles. For instance, wool fabric dyed with *A. nilotica* bark extract showed a decrease to 30% of its antioxidant activity after 20 washing cycles, while initially showing 87%. In this study, the use of mordants allowed for a slightly better (40%) antioxidant activity after washing cycles [51]. In another study, cotton and wool fabrics dyed with *Hibiscus* flower extract showed over 75% free radical scavenging activity with and without mordants after five washing cycles [87]. As observed for other functionalities, the use of mordants or crosslinkers also allowed for the obtention of better antioxidant activities [33,61,72,116]. Nevertheless, wool fabric dyed with an extract obtained from pineapple peel showed distinctly better antioxidant activity without a mordant when compared to that of wool dyed using a ferrous mordant [110].

These differences in textile dye efficacies require further research to achieve optimal treatment and dyeing conditions in order to maximize the functionalities of fabrics.

### 4.4. Flame Retardance

Flame-retardant treatment is used to reduce the risk of fire in textiles because they are quite flammable and capable of burning well. There are inorganic and organic flame-retardant compounds commercially available. Organic compounds are the most utilized as they can be applied to almost all textiles whereas inorganic compounds are mainly applied to wool fabrics [82]. The most used organic flame-retardant compounds are bromine-, chlorine-, phosphorus-, and nitrogen-containing compounds, but some of them are toxic or not eco-friendly [82]. Although numerous polyphenolic natural dyes are reported to provide functionalities such as those described in the previous sections, not enough attention has yet been given towards their application as flame retardants.

Few studies have reported the flame retardance functionality of fabrics dyed with polyphenolic extracts. The studies that reported this functionality evaluated flame-retardant properties through the limited oxygen index (LOI) in addition to vertical flammability tests according to the standardized methods GB/T 5454-1997 [145], ASTM D2863 [146], GB/T 5455-2014 [147] and ASTM D6413 [27,82,120,127,148]. Fabrics exhibiting LOI values higher than 25% are considered flame-retardant.

A natural dye obtained from *D. cirrhosa* tubers was able to provide silk fabric with flame retardance properties, even after 20 washing cycles (LOI higher than 28%). The flame retardance function provided by this dye was attributed to the presence of condensed tannins [82]. Tannins possess high chemical and thermal stability alongside low thermal conductivity due to their specific aromatic structure, which makes them suitable for providing textiles with flame retardance properties for various applications [82]. Tea stem waste extract was also able to provide silk fabric with good flame retardance properties (LOI of 25.6%). With metallic salt mordants, this property was slightly increased (LOI of 26.75%) [127]. This property was attributed to the polymerized products in tea stem extract and the formation of natural polyphenols/metal ions/silk fabric complexes [127]. Proanthocyanins from grape seeds were also able to impair silk fabric with durable flame retardance properties (LOI of 27%). This property was maintained after 20 washing cycles [27]. Wool fabric dyed with rice straw extract also showed flame retardance properties due to the presence of phenolic compounds in combination with different mordants (LOI of 27.5%). However, after 20 washing cycles, the LOI value decreased below 25% [120].

### 4.5. Other Functionalities

To a lesser extent, other functionalities are reported to textile fabrics dyed with phenolic dyes. For instance, highly hydrophobic fabrics were able to be fabricated by using nature-inspired polyphenol chemistry. Using tannic, ferulic, and caffeic acids for coating fabrics such as viscose and cotton enabled the loading of hydrophobic particles (i.e., silver nanoparticles, Fe (III), and DTM@Ti(OH)_4_) onto them [35,36,41]. These metal-organic systems coated the fabrics and affected their surface roughness, making the textiles hydrophobic. All of the fabrics revealed an excellent hydrophobic capacity even after several washing cycles (25 to 50 washing cycles). Other authors reported that lotus leaf extract containing polyphenols and flavonoids was able to enhance the hydrophobicity of dyed polyester yarns [91].

Insect resistance has also been reported. For instance, a polyphenolic extract obtained from mango seed kernel was able to provide wool fabric with insect repellence activity against larvae of *Tineola bisselliella*. These insects can digest keratin protein causing premature damage to wool-made fabrics [16]. In addition, orange peel extract was also able to provide linen fabric with insect resistance activity [105]. Moreover, anti-moth properties were reported in wool fabric dyed with *M. azedarach* bark extract against the larvae of the black carpet beetle (*Attagenus unicolor*) [97].

## 5. Potential Textile Industry Applications of Phenolic Dyes

Although the research referenced throughout this review has been conducted on a laboratory scale, several works state the potential of the obtained dyes to be used for specific materials or products on an industrial scale. For instance, the coating of jute fabric with different phenolic compounds showed excellent UV resistance and could be used in technical textiles outdoors, such as packing bags, ropes, and textile coverings, to increase their working life under sunlight and maintain their mechanical properties for longer [32]. The multifunctional viscose textiles prepared with tannic acid have potential applications for use in biomedical bandages or protective clothing for working in unsanitary and moist environments [35]. Xing et al. [41] suggested that the inclusion of natural polyphenols and DTM@Ti(OH)_4_ particles onto cotton fabric could have several applications in daily life, including usage in raincoats, sunscreen clothing, outdoor tents, curtains, waterproof cloth, and others. Furthermore, Zhou et al. [73] stated that the excellent UV protective, antibacterial, and antioxidant properties of *S. sebiferum* leaf extract and dyed wool fabric could be potentially exploited for the development of bioactive sutures, bandages, scaffolds, wound dressing, masks, and surgical gowns. In addition, cotton fabrics dyed with chestnut shell extract could be used for clothing materials, home textiles, and upholstery fabrics, while wool fabrics sustainably dyed with rice straw could be explored in a variety of textile applications including hospital textiles, outdoor textiles, and flame retardance apparels [67,120].

Different studies show that agri-food by-products and wastes provide adequate streams for the valorization of natural dyes, especially when applied on a niche scale [102]. Additionally, these studies have established a groundwork for future investigations where these and other raw materials and by-products rich in polyphenols could be researched and scaled up to be implemented at an industrial level for the production of functional and smart textiles.

## 6. Conclusions, Limitations, and Perspectives

Undeniably, there has been increased research in natural dyes rich in phenolic compounds for added-value textile applications. The changes in our climate and environment have raised the need for more functional textiles in terms of protection against a variety of factors. In addition, with the high amounts of toxic wastes generated by the textile industry, the need for more sustainable processes for dye obtention and their application has become imperative. Various studies have been conducted on the search for natural dyes rich in polyphenols from different sources, including by-products from the agri-food industry. While no natural dye is yet absolutely sustainable, several active measures are being taken to improve this aspect. For this reason, dyes from natural sources could be utilized on a larger scale as a real possible alternative to synthetic dyes. In addition to the sustainability aspect, these polyphenolic dyes provide significant functional properties to dyed fabrics such as antimicrobial, UV protective, antioxidant, flame-retardant, and insect-repellent.

Some limitations were also identified in the present study. For instance, it may have been susceptible to publication bias, where positive results were more likely to be published than negative ones. Additionally, studies that were not indexed in the searched databases could have gone unnoticed. Furthermore, although the majority of the dyes were obtained through more eco-friendly and sustainable processes, this review did not fully address the potential environmental impacts of using phenolic compounds from by-products as it focused primarily on their functional properties. In addition, this review also focused predominantly on the initial stages of dye obtention and textile processing, overlooking industrial applications and potential challenges in the final product.

Despite these limitations, specific conclusions can be drawn from this review article:

- By-products from the agri-food industries are an excellent source of multifunctional natural dyes rich in phenolic compounds.
- Despite the increased research in this field, the screening of different by-products must continue to better understand their potential.
- There is great potential for antimicrobial, UV protective, and antioxidant activities of polyphenolic dyes, while other activities such as flame retardance and insect repellency are also gaining more attention.
- Eco-friendly dyeing practices must continue to be implemented and improved upon to achieve more sustainable dyeing processes.
- Bio-sourced mordants help provide a truly sustainable dyeing solution by eliminating the use of metallic mordants, but metallic mordants are still the main players in the industry. Thus, in conjunction with new natural dyes, biomordants should also be a main target of research.
- All studies reported were performed on a laboratory scale. Scale-ups and implementation of these processes in industrial settings should be a goal to understanding their practical and economic viability.

## Data Availability

Data are contained within the article and Supplementary Materials.

## Supplementary Materials

The following supporting information can be downloaded at: https://www.mdpi.com/article/10.3390/ma16227248/s1, Table S1. List of journals and respective quartiles of the articles obtained in the literature research (Q1-28; Q2-16; Q3-5).

## References

[1] Agnhage T., Perwuelz A., Behary N. (2017). Towards Sustainable *Rubia tinctorum* L. Dyeing of Woven Fabric: How Life Cycle Assessment Can Contribute. J. Clean. Prod..

[2] Rahman S.S., Siddiqua S., Cherian C. (2022). Sustainable Applications of Textile Waste Fiber in the Construction and Geotechnical Industries: A Retrospect. Clean. Eng. Technol..

[3] Wang F., Gong J., Ren Y., Zhang J. (2018). Eco-Dyeing with Biocolourant Based on Natural Compounds. R. Soc. Open Sci..

[4] Shabbir M., Rather L.J., Shahid-ul-Islam, Bukhari M.N., Shahid M., Ali Khan M., Mohammad F. (2016). An Eco-Friendly Dyeing of Woolen Yarn by Terminalia Chebula Extract with Evaluations of Kinetic and Adsorption Characteristics. J. Adv. Res..

[5] Javaid R., Qazi U.Y. (2019). Catalytic Oxidation Process for the Degradation of Synthetic Dyes: An Overview. Int. J. Environ. Res. Public. Health.

[6] Jia Y., Liu B., Cheng D., Li J., Huang F., Lu Y. (2017). Dyeing Characteristics and Functionability of Tussah Silk Fabric with Oak Bark Extract. Text. Res. J..

[7] Yadav S., Tiwari K.S., Gupta C., Tiwari M.K., Khan A., Sonkar S.P. (2023). A Brief Review on Natural Dyes, Pigments: Recent Advances and Future Perspectives. Results Chem..

[8] Velusamy S., Roy A., Sundaram S., Kumar Mallick T. (2021). A Review on Heavy Metal Ions and Containing Dyes Removal Through Graphene Oxide-Based Adsorption Strategies for Textile Wastewater Treatment. Chem. Rec..

[9] Global Organic Textile Standard (GOTS) Version 4.0. www.global-standard.org.

[10] Danila A., Costea M., Profire L., Rimbu C.M., Baican M., Lupascu F., Tatarusanu S.M., Profire B.S., Muresan E.I. (2022). A Sustainable Approach to a Cleaner Production of Antimicrobial and Biocompatible Protein Fibers. Polymers.

[11] Albuquerque B.R., Heleno S.A., Oliveira M.B.P.P., Barros L., Ferreira I.C.F.R. (2021). Phenolic Compounds: Current Industrial Applications, Limitations and Future Challenges. Food Funct..

[12] Mark R., Lyu X., Lee J.J.L., Parra-Saldívar R., Chen W.N. (2019). Sustainable Production of Natural Phenolics for Functional Food Applications. J. Funct. Foods.

[13] Brudzyńska P., Sionkowska A., Grisel M. (2021). Plant-Derived Colorants for Food, Cosmetic and Textile Industries: A Review. Materials.

[14] Zeng P., Chen X., Qin Y.R., Zhang Y.H., Wang X.P., Wang J.Y., Ning Z.X., Ruan Q.J., Zhang Y.S. (2019). Preparation and Characterization of a Novel Colorimetric Indicator Film Based on Gelatin/Polyvinyl Alcohol Incorporating Mulberry Anthocyanin Extracts for Monitoring Fish Freshness. Food Res. Int..

[15] Grifoni D., Bacci L., Di Lonardo S., Pinelli P., Scardigli A., Camilli F., Sabatini F., Zipoli G., Romani A. (2014). UV Protective Properties of Cotton and Flax Fabrics Dyed with Multifunctional Plant Extracts. Dye. Pigment..

[16] Hassan M.M. (2021). Enhanced Insect-Resistance, UV Protection, and Antibacterial and Antistatic Properties Exhibited by Wool Fabric Treated with Polyphenols Extracted from Mango Seed Kernel and Feijoa Peel. RSC Adv..

[17] Gómez-Moreno H., Duran-Serra A., Prieto-Fuentes R., Álvarez del Castillo M.D., Macanás J., Carrillo-Navarrete F. (2023). Almond Skin, a Bio-Waste for Green Dyeing of Wool Fibres. Text. Res. J..

[18] Guo L., Yang Z.Y., Tang R.C., Yuan H. (2020). Bin Preliminary Studies on the Application of Grape Seed Extract in the Dyeing and Functional Modification of Cotton Fabric. Biomolecules.

[19] Rossi T., Silva P.M.S., De Moura L.F., Araújo M.C., Brito J.O., Freeman H.S. (2017). Waste from Eucalyptus Wood Steaming as a Natural Dye Source for Textile Fibers. J. Clean. Prod..

[20] Durazzo A., Lucarini M., Souto E.B., Cicala C., Caiazzo E., Izzo A.A., Novellino E., Santini A. (2019). Polyphenols: A Concise Overview on the Chemistry, Occurrence, and Human Health. Phytother. Res..

[21] Sun S.S., Xing T., Tang R.C. (2013). Simultaneous Coloration and Functionalization of Wool, Silk, and Nylon with the Tyrosinase-Catalyzed Oxidation Products of Caffeic Acid. Ind. Eng. Chem. Res..

[22] Pizzi A. (2021). Tannins Medical / Pharmacological and Related Applications: A Critical Review. Sustain. Chem. Pharm..

[23] Alonso C., Martí M., Martínez V., Rubio L., Parra J.L., Coderch L. (2013). Antioxidant Cosmeto-Textiles: Skin Assessment. Eur. J. Pharm. Biopharm..

[24] Zhou Q., Rather L.J., Mir S.S., Ali A., Rizwanul Haque Q.M., Li Q. (2022). Bio Colourants from the Waste Leaves of *Ginkgo biloba* L. Tree: Wool Dyeing and Antimicrobial Functionalization against Some Antibiotic-Resistant Bacterial Strains. Sustain. Chem. Pharm..

[25] Guinot P., Benonge I., Nicolett G., Gargadennec A., Andary C., Rapior S. (2007). Combined Dyeing and Antioxidative Properties of Some Plant By-Products. Acta Bot. Gall..

[26] Yadav R., Mathur P., Sheikh J. (2019). Antibacterial, UV Protective and Antioxidant Linen Obtained by Natural Dyeing with Henna. Cellul. Chem. Technol..

[27] Guo L., Yang Z.Y., Tang R.C., Yuan H. (2020). Bin Grape Seed Proanthocyanidins: Novel Coloring, Flame-Retardant, and Antibacterial Agents for Silk. ACS Sustain. Chem. Eng..

[28] Li H., Li Z., Liu Y., Li M. (2020). Advantages of *Scutellaria baicalensis* Extracts over Just Baicalin in the Ultrasonically Assisted Multi-Functional Treatment of Linen Fabrics. Cellulose.

[29] Li Y.D., Guan J.P., Tang R.C., Qiao Y.F. (2019). Application of Natural Flavonoids to Impart Antioxidant and Antibacterial Activities to Polyamide Fiber for Health Care Applications. Antioxidants.

[30] Hong K.H. (2015). Phenol Compounds Treated Cotton and Wool Fabrics for Developing Multi-Functional Clothing Materials. Fibers Polym..

[31] Su J., Noro J., Silva S., Fu J., Wang Q., Ribeiro A., Silva C., Cavaco-Paulo A. (2019). Antimicrobial Coating of Textiles by Laccase in Situ Polymerization of Catechol and P-Phenylenediamine. React. Funct. Polym..

[32] Dong A., Yu Y., Fan X., Wang Q., Cavaco-Paulo A. (2016). Enzymatic Coating of Jute Fabrics for Enhancing Anti-Ultraviolent Properties via in-Situ Polymerization of Polyhydric Phenols. J. Ind. Text..

[33] Hong K.H. (2016). Crosslinking Phenolic Compounds with Cotton Fabrics Using Succinic Acid to Develop Functional Clothing Materials. Fibers Polym..

[34] Ibrahim N.A., Eid B.M., El-Zairy E.M., Emam E., Barakat S. (2019). Environmentally Sound Approach for Imparting Antibacterial and UV-Protection Functionalities to Linen Cellulose Using Ascorbic Acid. Int. J. Biol. Macromol..

[35] Bu Y., Zhang S., Cai Y., Yang Y., Ma S., Huang J., Yang H., Ye D., Zhou Y., Xu W. (2019). Fabrication of Durable Antibacterial and Superhydrophobic Textiles via in Situ Synthesis of Silver Nanoparticle on Tannic Acid-Coated Viscose Textiles. Cellulose.

[36] Gu S., Yang L., Huang W., Bu Y., Chen D., Huang J., Zhou Y., Xu W. (2017). Fabrication of Hydrophobic Cotton Fabrics Inspired by Polyphenol Chemistry. Cellulose.

[37] Antunes R.S., Ferraz D., Garcia L.F., Thomaz D.V., Luque R., Lobón G.S., Gil E.d.S., Lopes F.M. (2018). Development of a Polyphenol Oxidase Biosensor from Jenipapo Fruit Extract (*Genipa americana* L.) and Determination of Phenolic Compounds in Textile Industrial Effluents. Biosensors.

[38] Anku W.W., Mamo M.A., Govender P.P. (2017). Phenolic Compounds in Water: Sources, Reactivity, Toxicity and Treatment Methods. Phenolic Compounds–Natural Sources, Importance and Applications.

[39] Shen L., Jiang J., Liu J., Fu F., Diao H., Liu X. (2022). Cotton Fabrics with Antibacterial and Antiviral Properties Produced by a Simple Pad-Dry-Cure Process Using Diphenolic Acid. Appl. Surf. Sci..

[40] Mongkholrattanasit R., Kryštůfek J., Wiener J., Studničková J. (2011). Properties of Wool and Cotton Fabrics Dyed with Eucalyptus, Tannin and Flavonoids. Fibres Text. East. Eur..

[41] Xing L., Wang B., Zhang Y., Yang H., Zhu X., Chen G., Xing T. (2021). Universal Fabrication of Superhydrophobic and UV Resistant Cotton Fabric with Polyphenols. Cellulose.

[42] Wang Y., Huang L., Wang P., Ran R., Zhang T. (2023). Silk Textile Finished with Natural Dyes and UV Resistance Agents from Agricultural Waste Aloe Vera Rinds. J. Text. Inst..

[43] Otaviano B.T.H., Sannomiya M., de Queiroz R.S., Sánchez A.A.C., Freeman H.S., Mendoza L.E.R., Veliz J.L.S., Leon M.M.G., Leo P., da Costa S.A. (2023). Natural Dye Extracted from Pomegranate Peel: Physicochemical Characterization, Dyeing of Cotton Fabric, Color Fastness, and Photoprotective Properties. Fibers Polym..

[44] Bouaziz A., Dridi D., Gargoubi S., Chelbi S., Boudokhane C., Kenani A., Aroui S. (2021). Analysis of the Coloring and Antibacterial Effects of Natural Dye: Pomegranate Peel. Coatings.

[45] Muresan E.I., Diaconu M., Zaharia C., Rosu G., Danila A., Pui A. (2020). Bioactive Textiles Obtained by Using Aqueous Extracts of Vine Leaves. Fibers Polym..

[46] Chen M.X., Haider M.K., Kim I.S., Lee J.S. (2023). Characterization of Antioxidant *Houttuynia Cordata* Extracts Loaded Polyurethane Nanofibers. Fash. Text..

[47] Raji Y., Nadi A., Chemchame Y., Mechnou I., Bouari A.E.L., Cherkaoui O., Zyade S. (2023). Eco-Friendly Extraction of Flavonoids Dyes from Moroccan (*Reseda luteola* L.), Wool Dyeing, and Antibacterial Effectiveness. Fibers Polym..

[48] Baaka N., Mahfoudhi A., Haddar W., Mhenni M.F., Mighri Z. (2017). Green Dyeing Process of Modified Cotton Fibres Using Natural Dyes Extracted from *Tamarix aphylla* (L.) Karst. Leaves. Nat. Prod. Res..

[49] Fernández-Ponce M.T., Medina-Ruiz E., Casas L., Mantell C., Martínez de la Ossa-Fernández E.J. (2018). Development of Cotton Fabric Impregnated with Antioxidant Mango Polyphenols by Means of Supercritical Fluids. J. Supercrit. Fluids.

[50] Chakraborty L., Pandit P., Roy Maulik S. (2020). Acacia Auriculiformis–A Natural Dye Used for Simultaneous Coloration and Functional Finishing on Textiles. J. Clean. Prod..

[51] Rather L.J., Akhter S., Padder R.A., Hassan Q.P., Hussain M., Khan M.A., Mohammad F. (2017). Colorful and Semi Durable Antioxidant Finish of Woolen Yarn with Tannin Rich Extract of Acacia Nilotica Natural Dye. Dye. Pigment..

[52] Alebeid O.K., Pei L., Elhassan A., Zhou W., Wang J. (2020). Cleaner Dyeing and Antibacterial Activity of Wool Fabric Using Henna Dye Modified with Acacia Nilotica Pods. Clean. Technol. Environ. Policy.

[53] Rather L.J., Shabbir M., Li Q., Mohammad F. (2019). Coloration, UV Protective, and Antioxidant Finishing of Wool Fabric Via Natural Dye Extracts: Cleaner Production of Bioactive Textiles. Environ. Prog. Sustain. Energy.

[54] Andriamanantena M., Razafimbelo F.F., Raonizafinimanana B., Cardon D., Danthu P., Lebeau J., Petit T., Caro Y. (2021). Alternative Sources of Red Dyes with High Stability and Antimicrobial Properties: Towards an Ecological and Sustainable Approach for Five Plant Species from Madagascar. J. Clean. Prod..

[55] Shabbir M., Mohammad F. (2018). Multifunctional AgNPs@Wool: Colored, UV-Protective and Antioxidant Functional Textiles. Appl. Nanosci..

[56] Saleem M.A., Nazir A., Nazir F., Ayaz P., Faizan M.Q., Usman M., Hussain T. (2019). Comparison of UV Protection Properties of Cotton Fabrics Treated with Aqueous and Methanolic Extracts of Solanum Nigrum and Amaranthus Viridis Plants. Photodermatol. Photoimmunol. Photomed..

[57] Rahman Liman M.L., Islam M.T., Repon M.R., Hossain M.M., Sarker P. (2021). Comparative Dyeing Behavior and UV Protective Characteristics of Cotton Fabric Treated with Polyphenols Enriched Banana and Watermelon Biowaste. Sustain. Chem. Pharm..

[58] Rehman A., Irfan M., Hameed A., Saif M.J., Qayyum M.A., Farooq T. (2022). Chemical-Free Dyeing of Cotton with Functional Natural Dye: A Pollution-Free and Cleaner Production Approach. Front. Environ. Sci..

[59] Wang P., Wu H., Zheng X., Bian L., Sun Y., Wang Z., Li C. (2022). High-Binding-Fastness Dye from Functional Extracts of Keemun Black Tea Waste for Dyeing Flax Fabric. Color. Technol..

[60] Zhang W., Yao J., Huang P., Xing S. (2020). Aqueous Extraction of Buckwheat Hull and Its Functional Application in Eco-Friendly Dyeing for Wool Fabric. Text. Res. J..

[61] Shahid-ul-Islam, Butola B.S., Roy A. (2018). Chitosan Polysaccharide as a Renewable Functional Agent to Develop Antibacterial, Antioxidant Activity and Colourful Shades on Wool Dyed with Tea Extract Polyphenols. Int. J. Biol. Macromol..

[62] Kim S. (2006). Dyeing Characteristics and UV Protection Property of Green Tea Dyed Cotton Fabrics -Focusing on the Effect of Chitosan Mordating Condition. Fibers Polym..

[63] Saini S., Gupta A., Singh N., Sheikh J. (2020). Functionalization of Linen Fabric Using Layer by Layer Treatment with Chitosan and Green Tea Extract. J. Ind. Eng. Chem..

[64] Muruganandham M., Sivasubramanian K., Velmurugan P., Suresh Kumar S., Arumugam N., Almansour A.I., Suresh Kumar R., Manickam S., Pang C.H., Sivakumar S. (2023). An Eco-Friendly Ultrasound Approach to Extracting Yellow Dye from Cassia Alata Flower Petals: Characterization, Dyeing, and Antibacterial Properties. Ultrason. Sonochem.

[65] Singh A., Sheikh J. (2022). Development of Mosquito Repellent, Antibacterial, Antioxidant and UV Protective Cotton Using a Novel Method of Azoic Dyeing with Terminalia Chebula. J. Nat. Fibers.

[66] Hong K.H. (2021). Sustainable Functional Finishing for Cotton Fabrics Using Chestnut Shell Extract. Cellulose.

[67] Hong K.H. (2023). Sustainable Functionalization for Cotton Fabrics by Printing with a Mixture of Chestnut Shell Extract and Alginate. Polym. Bull..

[68] Jose S., Pandit P., Pandey R. (2019). Chickpea Husk–A Potential Agro Waste for Coloration and Functional Finishing of Textiles. Ind. Crops Prod..

[69] Bai R., Yu Y., Wang Q., Yuan J., Fan X. (2016). Effect of Laccase on Dyeing Properties of Polyphenol-Based Natural Dye for Wool Fabric. Fibers Polym..

[70] Zhang B., Wang L., Luo L., King M.W. (2014). Natural Dye Extracted from Chinese Gall–The Application of Color and Antibacterial Activity to Wool Fabric. J. Clean. Prod..

[71] Lee Y.H., Hwang E.K., Baek Y.M., Kim H.-D. (2015). Deodorizing Function and Antibacterial Activity of Fabrics Dyed with Gallnut (*Galla chinensis*) Extract. Text. Res. J..

[72] Hassan M.M., Saifullah K. (2021). Sustainable Dyeing and Functionalization of Jute Fabric with a Chinese Sumac Gall-Derived Gallotannin Using Eco-Friendly Mordanting Agents. Cellulose.

[73] Zhou Q., Rather L.J., Ali A., Wang W., Zhang Y., Rizwanul Haque Q.M., Li Q. (2020). Environmental Friendly Bioactive Finishing of Wool Textiles Using the Tannin-Rich Extracts of Chinese Tallow (*Sapium sebiferum* L.) Waste/Fallen Leaves. Dye. Pigment..

[74] Rather L.J., Ali A., Zhou Q., Ganie S.A., Gong K., Rizwanul Haque Q.M., Li Q. (2020). Instrumental Characterization of Merino Wool Fibers Dyed with Cinnamomum Camphora Waste/Fallen Leaves Extract: An Efficient Waste Management Alternative. J. Clean. Prod..

[75] Gong K., Pan Y., Rather L.J., Wang W., Zhou Q., Zhang T., Li Q. (2019). Natural Pigment during Flora Leaf Senescence and Its Application in Dyeing and UV Protection Finish of Silk and Wool–A Case Study of Cinnamomum Camphora. Dye. Pigment..

[76] Rehman A., Ahmad A., Hameed A., Kiran S., Farooq T. (2021). Green Dyeing of Modified Cotton Fabric with Acalypha Wilkesiana Leave Extracts. Sustain. Chem. Pharm..

[77] Pinheiro M.N.C., Symochko L., Castro L.M. (2023). Valorization of Cork Industry By-Products as Sustainable Natural Dyes for Textiles. ACS Sustain. Chem. Eng..

[78] Silva P.M.d.S., Fiaschitello T.R., de Queiroz R.S., Freeman H.S., da Costa S.A., Leo P., Montemor A.F., da Costa S.M. (2020). Natural Dye from Croton Urucurana Baill. Bark: Extraction, Physicochemical Characterization, Textile Dyeing and Color Fastness Properties. Dye. Pigment..

[79] Maksimovic S., Tadic V., Zvezdanovic J., Zizovic I. (2021). Utilization of Supercritical CO_2_ in Bioactive Principles Isolation from Helichrysum Italicum and Their Adsorption on Selected Fabrics. J. Supercrit. Fluids.

[80] Souissi M., Guesmi A., Moussa A. (2018). Valorization of Natural Dye Extracted from Date Palm Pits (*Phoenix dactylifera*) for Dyeing of Cotton Fabric. Part 1: Optimization of Extraction Process Using Taguchi Design. J. Clean. Prod..

[81] Guesmi A., Ben Hamadi N. (2018). Study on Optimizing Dyeing of Cotton Using Date Pits Extract as a Combined Source of Coloring Matter and Bio-Mordant. Nat. Prod. Res..

[82] Yang T.T., Guan J.P., Tang R.C., Chen G. (2018). Condensed Tannin from Dioscorea Cirrhosa Tuber as an Eco-Friendly and Durable Flame Retardant for Silk Textile. Ind. Crops Prod..

[83] Haddar W., Elksibi I., Meksi N., Mhenni M.F. (2014). Valorization of the Leaves of Fennel (*Foeniculum vulgare*) as Natural Dyes Fixed on Modified Cotton: A Dyeing Process Optimization Based on a Response Surface Methodology. Ind. Crops Prod..

[84] Zhou Y., Tawiah B., Wang L., Li Q. (2022). Enhancing the Affinity and Adsorption Efficiency of Glochidion Ericarpum Champ Leave Extract to Cotton for Colouristic and Functional Properties Integrating Trimethyl Chitosan and Ultrasonic Technique. Ind. Crops Prod..

[85] Li Q., Zhang N., Ni L., Wei Z., Quan H., Zhou Y. (2021). One-Pot High Efficiency Low Temperature Ultrasonic-Assisted Strategy for Fully Bio-Based Coloristic, Anti-Pilling, Antistatic, Bioactive and Reinforced Cashmere Using Grape Seed Proanthocyanidins. J. Clean. Prod..

[86] Pandit P., Jose S., Pandey R. (2021). Groundnut Testa: An Industrial Agro-Processing Residue for the Coloring and Protective Finishing of Cotton Fabric. Waste Biomass Valorization.

[87] Rehan M., Ibrahim G.E., Mashaly H.M., Hasanin M., Rashad H.G., Mowafi S. (2022). Simultaneous Dyeing and Multifunctional Finishing of Natural Fabrics with Hibiscus Flowers Extract. J. Clean. Prod..

[88] Safapour S., Rather L.J., Safapour R., Mir S.S. (2023). Valorization of Bio-Colorants Extracted from *Hypercium scabrum* L. Plant for Sustainable and Ecological Coloration of Wool Yarns. Heliyon.

[89] Rani N., Jajpura L., Butola B.S. (2022). Sustainable Coloration of Protein Fibers Using Kalanchoe-Pinnata Leaf Extract. J. Nat. Fibers.

[90] Lee Y.H., Kim A.L., Park Y.G., Hwang E.K., Baek Y.M., Cho S., Kim H.-D. (2018). Colorimetric Assay and Deodorizing/Antibacterial Performance of Natural Fabrics Dyed with Immature Pine Cone Extract. Text. Res. J..

[91] Zhu Q., Zhang C., Zhu N., Gong J., Zhou Z., Sheng D., Zhou S., Wang X., Fu Z., Xia L. (2023). Preparation of Polyester Yarns with Bright Color and Enhanced Hydrophobicity Using Lotus Leaf Powders. Ind. Crops Prod..

[92] Dong Y., Gu J., Wang P., Wen H. (2019). Developed Functionalization of Wool Fabric with Extracts of Lycium Ruthenicum Murray and Potential Application in Healthy Care Textiles. Dye. Pigment..

[93] Shahmoradi Ghaheh F., Mortazavi S.M., Alihosseini F., Fassihi A., Shams Nateri A., Abedi D. (2014). Assessment of Antibacterial Activity of Wool Fabrics Dyed with Natural Dyes. J. Clean. Prod..

[94] Liman M.L.R., Islam M.T., Hossain M.M., Sarker P., Repon M.R. (2021). Environmentally Benign Dyeing Mechanism of Knitted Cotton Fabric with Condensed and Hydrolyzable Tannin Derivatives Enriched Bio-Waste Extracts. Environ. Technol. Innov..

[95] Sanchez-Sanchez J., Fernández-Ponce M.T., Casas L., Mantell C., de la Ossa E.J.M. (2017). Impregnation of Mango Leaf Extract into a Polyester Textile Using Supercritical Carbon Dioxide. J. Supercrit. Fluids.

[96] Vuthiganond N., Nakpathom M., Mongkholrattanasit R. (2018). Metal-Free Dyeing of Cotton Fabric Using Mangrove Bark Polyphenols via Azoic Dyeing. Fibers Polym..

[97] Tian Y., Lu Y., Zhang Y., Hou X., Zhang Y. (2022). Extraction and Characterization of Natural Colorant from Melia Azedarach Bark and Its Utilization in Dyeing and Finishing of Wool. Sustain. Chem. Pharm..

[98] Räisänen R., Primetta A., Toukola P., Fager S., Ylänen J. (2023). Biocolourants from Onion Crop Side Streams and Forest Mushroom for Regenerated Cellulose Fibres. Ind. Crops Prod..

[99] Ma M., Li R., Du Y., Tang Z., Zhou W. (2013). Analysis of Antibacterial Properties of Naturally Colored Cottons. Text. Res. J..

[100] Meksi N., Haddar W., Hammami S., Mhenni M.F. (2012). Olive Mill Wastewater: A Potential Source of Natural Dyes for Textile Dyeing. Ind. Crops Prod..

[101] Volpi C., Bartolini D., Brighenti V., Galli F., Tiecco M., Pellati F., Clementi C., Sardella R. (2021). Antioxidant Power on Dermal Cells by Textiles Dyed with an Onion (*Allium cepa* L.) Skin Extract. Antioxidants.

[102] Grande R., Räisänen R., Dou J., Rajala S., Malinen K., Nousiainen P.A., Österberg M. (2023). In Situ Adsorption of Red Onion (*Allium cepa*) Natural Dye on Cellulose Model Films and Fabrics Exploiting Chitosan as a Natural Mordant. ACS Omega.

[103] Pucciarini L., Ianni F., Petesse V., Pellati F., Brighenti V., Volpi C., Gargaro M., Natalini B., Clementi C., Sardella R. (2019). Onion (*Allium cepa* L.) Skin: A Rich Resource of Biomolecules for the Sustainable Production of Colored Biofunctional Textiles. Molecules.

[104] Hou X., Chen X., Cheng Y., Xu H., Chen L., Yang Y. (2013). Dyeing and UV-Protection Properties of Water Extracts from Orange Peel. J. Clean. Prod..

[105] Rehan M., Abdel-Wahed N.A.M., Farouk A., El-Zawahry M.M. (2018). Extraction of Valuable Compounds from Orange Peel Waste for Advanced Functionalization of Cellulosic Surfaces. ACS Sustain. Chem. Eng..

[106] Boussoum M.O., Ali-Nehari A., Ouldmokhtar R., George B. (2021). Characterization of Extracts from Papaver Rhoeas and Potential Valorization of These Extracts in Dyeing Applications. Turk. J. Chem..

[107] Rehan M., Elshemy N.S., Haggag K., Montaser A.S., Ibrahim G.E. (2020). Phytochemicals and Volatile Compounds of Peanut Red Skin Extract: Simultaneous Coloration and in Situ Synthesis of Silver Nanoparticles for Multifunctional Viscose Fibers. Cellulose.

[108] Pandey R., Patel S., Pandit P., Nachimuthu S., Jose S. (2018). Colouration of Textiles Using Roasted Peanut Skin- an Agro Processing Residue. J. Clean. Prod..

[109] Moussa I., Ghezal I., Sakli F. (2023). Valorization of Pelargonium Graveolens L’Hér. Hydrodistillation Solid Waste as Natural Dye for Wool Fabrics. J. Nat. Fibers.

[110] Sheikh J., Agrawal A., Garg H., Agarwal A., Mathur P. (2019). Functionalization of Wool Fabric Using Pineapple Peel Extract (PPE) as a Natural Dye. AATCC J. Res..

[111] Inprasit T., Pukkao J., Lertlaksameephan N., Chuenchom A., Motina K., Inprasit W. (2020). Green Dyeing and Antibacterial Treatment of Hemp Fabrics Using Punica Granatum Peel Extracts. Int. J. Polym. Sci..

[112] Sadeghi-Kiakhani M., Tehrani-Bagha A.R., Gharanjig K., Hashemi E. (2019). Use of Pomegranate Peels and Walnut Green Husks as the Green Antimicrobial Agents to Reduce the Consumption of Inorganic Nanoparticles on Wool Yarns. J. Clean. Prod..

[113] Baseri S. (2020). Eco-Friendly Production of Anti-UV and Antibacterial Cotton Fabrics via Waste Products. Cellulose.

[114] Zhang W., Wang X., Weng J., Liu X., Qin S., Li X., Gong J. (2022). Eco-Dyeing and Functional Finishing of Wool Fabric Based on *Portulaca Oleracea* L. as Colorant and Musa Basjoo as Natural Mordant. Arab. J. Chem..

[115] Yin Y., Jia J., Wang T., Wang C. (2017). Optimization of Natural Anthocyanin Efficient Extracting from Purple Sweet Potato for Silk Fabric Dyeing. J. Clean. Prod..

[116] Koh E., Hong K.H. (2017). Functional Fabric Treatment Using Tannic Acid and Extract from Purple-Fleshed Sweet Potato. Text. Res. J..

[117] Yongchun D., Wang L., Yan Y., Gu J. (2023). Optimized Preparation and Dyeing of Pterocarpus Santalinus Waste Extract for Enhancing Healthy and Environmental Care Performance of Wool Fabric. J. Text. Inst..

[118] Cerempei A., Mureşan E.I., Cimpoeşu N., Carp-Cărare C., Rimbu C. (2016). Dyeing and Antibacterial Properties of Aqueous Extracts from Quince (*Cydonia oblonga*) Leaves. Ind. Crops Prod..

[119] El Ksibi I., Slama R.B., Faidi K., Ticha M.B., M’henni M.F. (2015). Mixture Approach for Optimizing the Recovery of Colored Phenolics from Red Pepper (*Capsicum annum* L.) by-Products as Potential Source of Natural Dye and Assessment of Its Antimicrobial Activity. Ind. Crops Prod..

[120] Kadam S., Sharma A., ul-Islam S., Bramhecha I., Sheikh J. (2020). Utilization of Rice Straw as a Source of Biomolecules for Sustainable Multifunctional Finishing Vis a Vis Dyeing of Wool. J. Nat. Fibers.

[121] Lachguer K., Boudadi I., Fayzi L., El Merzougui S., El Bouchti M., Cherkaoui O., Serghini M.A. (2023). Natural Extraction of Dyes from Saffron “Crocus Sativus L” Flower Waste, Cotton Dyeing, and Antioxidant Effectiveness. Pollution.

[122] Baseri S. (2023). Agricultural Crop of Scrophularia Striata as a New Dye for Eco-Friendly Dyeing and Bioactive Finishing of Handwoven Piles. Sustain. Chem. Pharm..

[123] Hou X., Fang F., Guo X., Wizi J., Ma B., Tao Y., Yang Y. (2017). Potential of Sorghum Husk Extracts as a Natural Functional Dye for Wool Fabrics. ACS Sustain. Chem. Eng..

[124] Wizi J., Wang L., Hou X., Tao Y., Ma B., Yang Y. (2018). Ultrasound-Microwave Assisted Extraction of Natural Colorants from Sorghum Husk with Different Solvents. Ind. Crops Prod..

[125] Xia W., Li Z., Tang Y., Li Q. (2023). Sustainable Recycling of Café Waste as Natural Bio Resource and Its Value Adding Applications in Green and Effective Dyeing/Bio Finishing of Textile. Sep. Purif. Technol..

[126] Fang J., Meng C., Zhang G. (2022). Agricultural Waste of Ipomoea Batatas Leaves as a Source of Natural Dye for Green Coloration and Bio-Functional Finishing for Textile Fabrics. Ind. Crops Prod..

[127] Cheng T.H., Liu Z.J., Yang J.Y., Huang Y.Z., Tang R.C., Qiao Y.F. (2019). Extraction of Functional Dyes from Tea Stem Waste in Alkaline Medium and Their Application for Simultaneous Coloration and Flame Retardant and Bioactive Functionalization of Silk. ACS Sustain. Chem. Eng..

[128] Amutha K., Grace Annapoorani S., Sudhapriya N. (2020). Dyeing of Textiles with Natural Dyes Extracted from *Terminalia arjuna* and *Thespesia Populnea* Fruits. Ind. Crops Prod..

[129] Zaharia C., Diaconu M., Muresan E.I., Danila A., Popescu A., Rosu G. (2020). Bioactive Emulsions with Beneficial Antimicrobial Application in Textile Material Production. Cellulose.

[130] Ghaheh F.S., Nateri A.S., Mortazavi S.M., Abedi D., Mokhtari J. (2012). The Effect of Mordant Salts on Antibacterial Activity of Wool Fabric Dyed with Pomegranate and Walnut Shell Extracts. Color. Technol..

[131] Sagar N.A., Pareek S., Sharma S., Yahia E.M., Lobo M.G. (2018). Fruit and Vegetable Waste: Bioactive Compounds, Their Extraction, and Possible Utilization. Compr. Rev. Food Sci. Food Saf..

[132] Ben-Othman S., Jõudu I., Bhat R. (2020). Bioactives from Agri-Food Wastes: Present Insights and Future Challenges. Molecules.

[133] FAO (Food and Agriculture Organisation of the United Nations) Food Wastage Footprint Impacts on Natural Resources. https://www.fao.org/3/i3347e/i3347e.pdf.

[134] Gómez-Mejía E., Rosales-Conrado N., León-González M.E., Madrid Y. (2019). Citrus Peels Waste as a Source of Value-Added Compounds: Extraction and Quantification of Bioactive Polyphenols. Food Chem..

[135] Çam M., Içyer N.C., Erdoǧan F. (2014). Pomegranate Peel Phenolics: Microencapsulation, Storage Stability and Potential Ingredient for Functional Food Development. LWT.

[136] (2019). Test Method for Antibacterial Finishes on Textile Materials: Assessment of.

[137] (2016). Antimicrobial Activity Assessment of Textile Materials: Agar Plate.

[138] (2020). Standard Test Method for Determining the Antimicrobial Activity of Antimicrobial Agents Under Dynamic Contact Conditions.

[139] (2013). Textiles—Determination of Antibacterial Activity of Textile Products.

[140] (2008). Textiles-Evaluation for Antibacterial Activity-Part 3: Shake Flask Method.

[141] (2020). Transmittance or Blocking of Erythemally Weighted Ultraviolet Radiation through Fabrics.

[142] (2009). Textiles—Evaluation for Solar Ultraviolet Radiation Protective Properties.

[143] (1996). Sun Protective Clothing–Evaluation and Classification.

[144] (2001). Textiles—Solar UV Protective Properties—Part 1: Method of Test for Apparel Fabrics.

[145] (1997). Textiles–Burning Behavior—Oxygen Index Method.

[146] (2023). Standard Test Method for Measuring the Minimum Oxygen Concentration to Support Candle-Like Combustion of Plastics (Oxygen Index).

[147] (2014). Textile—Burning Behavior—Determination of Damaged Length, Afterglow Time and Afterflame Time of Vertically Oriented Specimens.

[148] (2022). Standard Test Method for Flame Resistance of Textiles (Vertical Test).

