# Supplementary material for: Phenolic Compounds from By-Products for Functional Textiles
Source: Materials (Basel). 2023 Nov 20;16(22):7248. doi: 10.3390/ma16227248 (PMC10672813; doi:10.3390/ma16227248)
Supplement: Supplementary file 1 [file materials-16-07248-s001.zip › materials-2707737-supplementary.pdf]

Table S1. List of journals and respective quartile of the articles obtained in the literature research (Q1 - 28; Q2 - 16; Q3 - 5).

Q1	Q2	Q3
**Journal (nº of articles cited)**	**Journal (nº of articles cited)**	**Journal (nº of articles cited)**
ACS Omega (1)	Acta Botanica Gallica (1)	Cellulose Chemistry and Technology (1)
ACS Sustainable Chemistry and Engineering (4)	Applied Nanoscience (1)	Coloration Technology (2)
Antioxidants (2)	Coatings (1)	Fibres and Textiles in Eastern Europe (1)
Applied Surface Science (1)	Environmental Progress and Sustainable Energy (1)	Pollution (1)
Arabian Journal of Chemistry (1)	Fibers and Polymers (8)	Turkish Journal of Chemistry (1)
Biomolecules (1)	International Journal of Polymer Science (1)	
Cellulose (9)	Journal of Industrial Textiles (1)	
Clean Technologies and Environmental Policy (1)	Journal of Natural Fibers (4)	
Dyes and Pigments (6)	Journal of the Textile Institute (2)	
Environmental Technology and Innovation (1)	Materials Today: Proceedings (1)	
European Journal of Pharmaceutics and Biopharmaceutics (1)	Natural Product Research (2)	
Fashion and Textiles (1)	Photodermatology Photoimmunology and Photomedicine (1)	
Frontiers in Environmental Science (1)	Polymer Bulletin (1)	
Heliyon (1)	RSC Advances (1)	
Industrial and Engineering Chemistry Research (1)	Textile Research Journal (7)	
Industrial Crops and Products (12)	Waste and Biomass Valorization (1)	
International Journal of Biological Macromolecules (3)		
Journal of Advanced Research (1)		
Journal of Cleaner Production (14)		
Journal of Industrial and Engineering Chemistry (1)		
Journal of Supercritical Fluids (3)		
Molecules (1)		
Polymers (1)		
Reactive and Functional Polymers (1)		
Royal Society Open Science (1)		
Separation and Purification Technology (1)		
Sustainable Chemistry and Pharmacy (5)		
Ultrasonics Sonochemistry (1)		
**Total citations: 77**	**Total citations: 34**	**Total citations: 6**
